# Two New Antibiotic Pyridones Produced by a Marine Fungus, *Trichoderma* sp. Strain MF106

**DOI:** 10.3390/md12031208

**Published:** 2014-03-06

**Authors:** Bin Wu, Vanessa Oesker, Jutta Wiese, Rolf Schmaljohann, Johannes F. Imhoff

**Affiliations:** 1 Kieler Wirkstoff-Zentrum am GEOMAR Helmholtz Zentrum für Ozeanforschung Kiel, Kiel 24105, Germany; E-Mails: wubin@zju.edu.cn (B.W.); vlutz@geomar.de (V.O.); jwiese@geomar.de (J.W.); rschmaljohann@geomar.de (R.S.); 2 Ocean College, Zhejiang University, Hangzhou 310058, China

**Keywords:** pyridones, antibiotic, *Trichoderma*, marine fungus

## Abstract

Two unusual pyridones, trichodin A (**1**) and trichodin B (**2**), together with the known compound, pyridoxatin (**3**), were extracted from mycelia and culture broth of the marine fungus, *Trichoderma* sp. strain MF106 isolated from the Greenland Seas. The structures of the new compounds were characterized as an intramolecular cyclization of a pyridine basic backbone with a phenyl group. The structure and relative configuration of the new compounds were established by spectroscopic means. The new compound **1** and the known compound **3** showed antibiotic activities against the clinically relevant microorganism, *Staphylococcus epidermidis*, with IC_50_ values of 24 μM and 4 μM, respectively.

## 1. Introduction

Marine habitats provide unique ecological niches for fungi, which are of great interest as promising sources of new biologically active products. Since marine fungi live in a biologically competitive environment with unique physical and chemical parameters, such as pH, temperature, pressure, oxygen, light, nutrients and salinity, the chemical diversity of the secondary metabolites and their range of applications from marine fungi is high [1,2,3,4,5,6,7]. Pyridone alkaloids characterized by a pyridine and a mono sesquiterpene in their structures are important microbial secondary metabolites [8]. Pyridoxatin **3**, a potent free radical scavenger with activity 20 times as active as vitamin E, was first reported by Teshima *et al*. [9]. It has been synthesized by Snider’s group, because of the structural uniqueness and interesting bioactivity [10]. 8-Methyl pyridoxatin was reported to induce erythropoietin in human cells [11]. Fungi of the genus, *Trichoderma* (teleomorph *Hypocrea*, class Sordariomycetes, order Hypocreales) are widespread in both terrestrial and marine environments. The genus comprises approximately 150 species [12]. They are frequently found on decaying wood and in soil, as well as in marine sediments, marine sponges and mangrove forests [13]. Members of *Trichoderma* living in the rhizosphere of plants are seen as opportunistic plant symbionts and are beginning to be used in reasonably large quantities in plant agriculture, both for disease control and yield increases. The ability of Trichoderma sp. strains to parasitize or even prey on other fungi is widely used for the biological control of phytopathogenic fungi. Studies of mycoparasitism also have demonstrated that these fungi produce a rich mixture of antifungal enzymes, including chitinases and β-1,3 glucanases [14]. *Trichoderma longibrachiatum* is known as an opportunistic pathogen of immunocompromised mammals, including humans, and some species are common indoor contaminants [15,16]. Marine representatives of the genus *Trichoderma* produce a variety of bioactive metabolites [17], which include the antimycobacterial aminolipopeptide trichoderins [18], the antifungal, trichodermaketone A [13], the cytotoxic dipeptide, trichodermamide B [19], as well as antibacterial tetrahydroanthraquinone and xanthone derivatives [20].

In this study, two new pyridones were identified in cultures of the marine fungal strain, MF106.

## 2. Results and Discussion

### 2.1. Identification of Strain MF106

**Figure 1:**
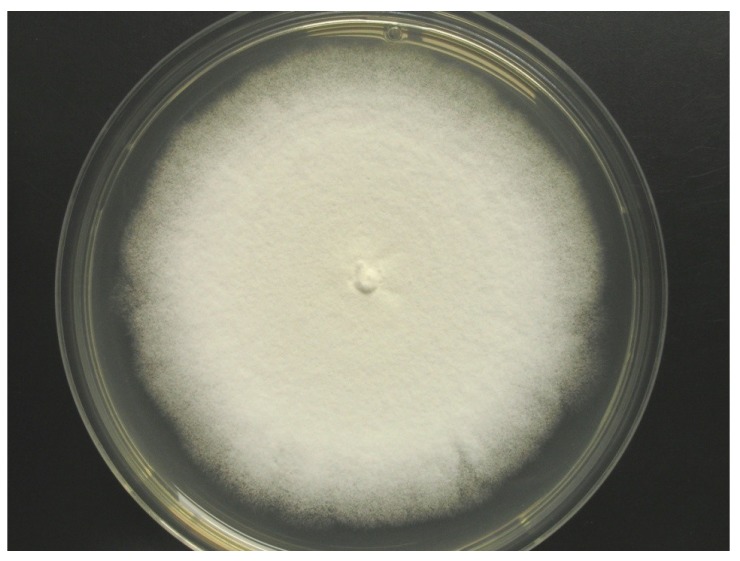
Colony of *Trichoderma* sp. strain MF106 on WSP medium.

**Figure 2:**
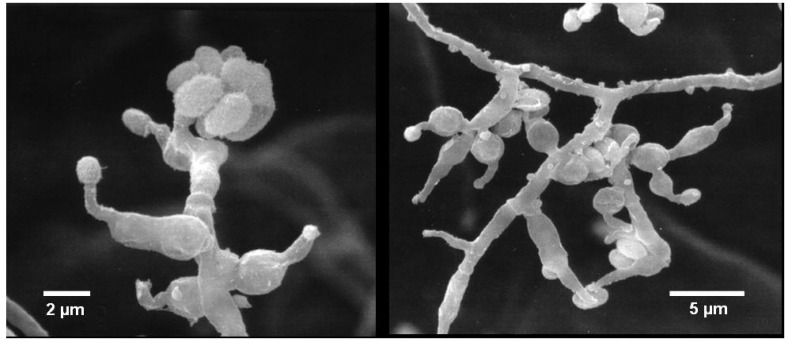
Scanning electron micrographs of *Trichoderma* sp. strain MF106, showing conidiophores with phialides producing rough-walled conidia at the end.

Colonies on WSP30 medium grew slowly and attained a diameter of 16 mm within seven days of incubation at 26 °C. They were pale white to greenish-white with a yellow back side (Figure 1). Conidia were produced only sparsely on WSP medium, as well as on further media (data not shown). The conidiophores were irregularly branched, divergent, bearing flask-shaped phialides with distinct necks. The conidia were colorless, rough-walled, ellipsoid-cylindrical, 4.5–5.0 × 2.0–2.2 µm in size and held in a mucilaginous drop at the end of each phialide (Figure 2). Strain MF106 showed morphological features typical of *Trichoderma sp*. Since the genus, *Trichoderma*, contains about 200 species [21], which are very difficult to differentiate by morphological criteria, it was not possible to classify strain MF106 on the species level.

### 2.2. Structural Elucidation

**Figure 3:**
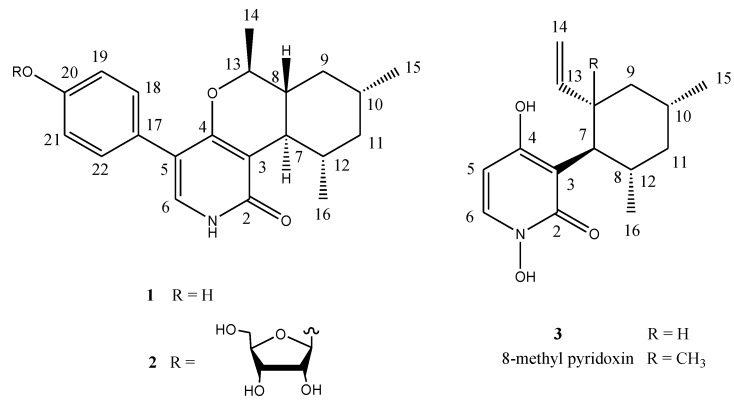
The structures of Compounds **1**–**3**.

Organic extracts of the cultured mycelia and broth of strain MF106 were subjected to repeated column chromatography on preparative HPLC (C18), to afford the two new compounds, **1** and **2**, and the known pyridoxine (**3**) [9] (Figure 3). Pyridoxatin (**3**) was the major component, whereas Compounds **1** and **2** were isolated as minor components.

Compound **1** was obtained as a brown powder. The HR-TOF-MS exhibited an ion peak at *m/z* 362.1751 [M + Na]^+^ (calcd. 362.1727), indicating that the molecular formula was C_21_H_25_NO_3_ with ten degrees of unsaturation. The IR spectrum revealed the presence of hydroxy groups and an aromatic ring characterized by absorptions at a *ν*_max_ of 3105 and 1614 cm^−1^, respectively. The phenolic nature of the compound was indicated by its characteristic color reactions (FeCl_3_: purple; phosphomolybdic acid reagent: deep blue).

**Figure 4:**
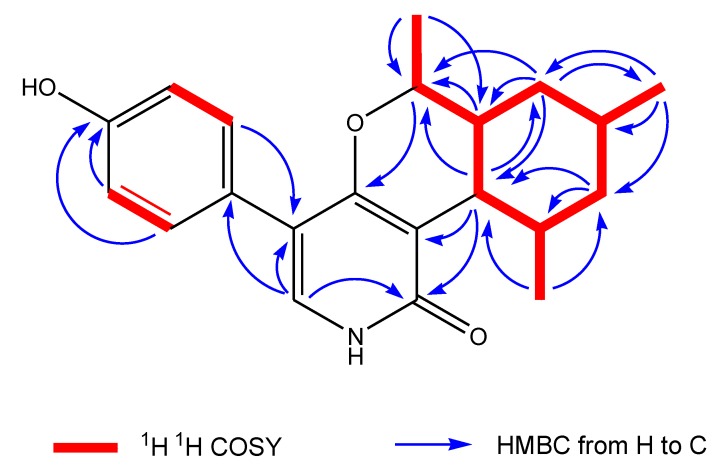
Key ^1^H ^1^H COSY and HMBC correlations of Compound **1**.

The ^13^C NMR spectrum showed the presence of 14 signals for pyridoxatin, including the pyridone skeleton and a cyclohexane substructure, with the remaining four resonances corresponding to a 1,4-disubstituted aromatic moiety. The presence of a 3,4,5-trisubstituted pyridin-2(1*H*)-one was supported by both ^1^H and ^13^C NMR data. The only pyridone proton signal at δ_H_ 7.13 showed a doublet with a small coupling constant of 0.5 Hz, attributing this proton to be adjacent to the NH of the pyridone ring and positioning a hydrogen on the pyridone nitrogen instead of a N-OH group in pyridoxatin (**3**). The diagnostic HMBC correlation from the pyridone doublet at δ_H_ 7.13 (d, *J* = 0.5 Hz, H-6) to the carbonyl at δ_C_ 165.9 (s, C-2) revealed a pyridin-2(1*H*)-one. The upfield region of the ^1^H ^1^H COSY spectrum of **1** demonstrated the presence of one proton spin systems connecting all the alkylic protons and consisting of four methylene protons, four methine protons, one oxygenated methine and three groups of methyl protons. The methine proton triplet at δ_H_ 2.29 (d, *J* = 10.1 Hz, H-7) coupled to the methine proton multiplet at δ_H_ 1.75 (m, H-12), which, in turn, coupled to a methyl proton doublet at δ_H_ 1.15 (d, *J* = 6.8, Me-16) and one methylene proton at δ_H_ 1.07 (dd, *J* = 24.8, 11.8 Hz, H-11α). This methylene proton was also coupled to its geminal partner at δ_H_ 1.80 (*br* d, *J* = 13.2 Hz, H-11β) and to the methine proton at δ_H_ 1.70 (m, H-10), which, in turn, coupled to the methyl doublets at δ_H_ 1.02 (d, *J* = 6.6 Hz, Me-15) and a pair of most upfield methylene protons at δ_H_ 0.89 (dd, *J* = 23.9, 12.1 Hz, H-9α) and a proton at δ_H_ 1.89 (*br* d, *J* = 14.9 Hz, H-9β), respectively. The proton signal at δ_H_ 1.54 (ddd, *J* = 23.9, 10.2, 2.6 Hz, H-8) showed cross peaks with methylene protons of H-9, the methine proton signal of H-7 and the oxymethine proton at δ_H_ 3.72 (m, H-13) coupled with methyl doublets at δ_H_ 1.31 (d, *J* = 6.3 Hz, Me-14). A sequence of cyclohexane substructure of H-7/H-8/H-9_2_/H-10/H-11_2_/H-12/H-7 with two Me groups at C-10 and C-12 was deduced from the above ^1^H ^1^H COSY analyses (Figure 4, Supplementary Information, Figures S4 and S5).

The NMR data (Table 1) in the upfield region of **1** were similar to those of the known pyridone compound, pyridoxatin (**3**), isolated from the same fungus, except that a doublet methyl protons signal (δ_H_ 1.31, (d, *J* = 6.3 Hz), Me-14) and oxygenated methine proton at δ_H_ 3.72 (m, H-13) were added in **1** [9]. In the downfield region of the NMR spectra of **1**, the terminal double bond signals were missing, when compared to the NMR data of pyridoxatin. These differences suggested that the terminal double bond at the C-8 of the cyclohexane ring transformed to a –OCH-CH_3_ unit, which was confirmed by the observation of HMBC correlations from Me-14 to C-8 and from methylene H-9 to the oxygenated C-13. The connectivity between the pyridin-2(1*H*)-one unit and the cyclohexane substructure were realized by detailed analyses of ^1^H–^13^C long-range correlations. The HMBC correlations from the methine proton triplet at δ_H_ 2.29 (d, *J* = 10.1 Hz, H-7) in the cyclohexane ring to the carbonyl carbon signal at δ_C_ 169.9 (s, C-2) and the carbon signal at δ_C_ 113.3 (s, C-3) in the pyridone unit indicated that the cyclohexane was linked at C-3. The oxymethine proton at δ_H_ 3.72 (m, H-13) exhibited a diagnostic long-range correlation with an aromatic carbon signal at δ_C_ 165.9 (s, C-4), revealing that an oxygen atom bridge was formed between C-4 at the pyridone unit and C-13 at the side chain of the cyclohexane unit. It is suggested that the oxygen in the –OCH-CH3 unit mentioned is the same one in pyridin-2(1*H*)-one unit of pyridoxatin (**3**) that ends up incorporated into the ring in compound **1** (Figure 3). The analyses of 1D and 2D NMR data completed the elucidation of a condensed ring system of pyridine, pyran and cyclohexane (Figure 4).

The formula indicated that six carbons remain to be assigned to the molecule of **1**. When the downfield region of the NMR spectra of **1** is compared with that of pyridoxatin (**3**), two additional sets of equivalent aromatic protons at δ_H_ 7.25 (d, *J* = 8.8 Hz, H-18, H-22) and δ_H_ 6.80 (d, *J* = 8.8 Hz, H-19, H-21) and four aromatic carbons at δ_C_ 126.6 (s, C-17),131.3 (d, C-18/22), 115.9 (d, C-19/21) and 157.9 (s, C-20) were observed, whereas one of the aromatic protons of the pyridone ring was absent (Table 1). The remaining four aromatic carbon and signals represented a *para*-substituted phenol, whose linkage position was assigned by the analyses of HMBC correlations. HMBC correlations from the only proton of H-6 at the pyridone unit to the aromatic C-17 and C-18/22 and from the equivalent aromatic protons of H-18/22 to the pyridone C-5 positioned the phenol at C-5. These 1D and 2D NMR analyses permitted the complete assignment of the planar structure of **1**, as shown in Figure 3. The proton signal of H-7 exhibited a triplet with a coupling constant of 10.1 Hz, suggestive of a 7,8-*trans* ring fusion between the pyran and cyclohexenyl rings.

**Figure 5:**
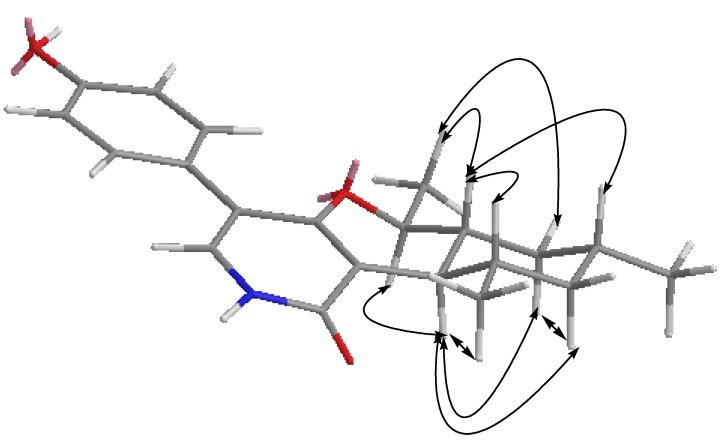
Key NOESY (nuclear Overhauser enhancement spectroscopy) correlations of Compound **1**.

Two sets of 1,3-diaxial NOESY correlations were observed (Figure 5, Supplementary Information, Figures S10 and S11). The 1,3-diaxial NOESY cross peaks of H-7α/H-9α, H-7α/H-11α and H-9α/H-11α revealed a cyclohexenyl boat ring in Molecule **1**. Additional 1,3-diaxial NOESY cross peaks of H-8β/H-12β and H-8β/H-10β indicated that two methyls at C-10 and C-12 were equatorial. The *trans* ring fusion between the pyran chair and cyclohexenyl boat rings were confirmed by the above 1,3-diaxial NOESY correlations. The proton signal of Me-14 showed NOESY correlations with H-8β and H-9β, assigning the Me-14 as β-oriented. This inference was confirmed by the diagnostic NOESY correlation of H-7α/H-13α. Thus, Compound **1** was elucidated as a new pyridone derivative. It is given the trivial name, trichodin A.

**Table 1:** NMR data for Compounds **1** (500 MHz), **2** and **3** (600 MHz) in CD_3_OD.

Position	1		2		3	
	δ_C_^a, b^, type	δ_H_^c^, multiplicities (*J* in Hz)	δ_C_^a, b^, type	δ_H_^c^, multiplicities (*J* in Hz)	δ_C_^a, b^, type	δ_H_^c^, multiplicities (*J* in Hz)
2	165.9, C	-	165.8, C	-	160.4, C	-
3	113.0, C	-	113.0, C	-	114.7, C	-
4	165.9, C	-	166.0, C	-	164.0, C	-
5	117.6, C	-	117.2, C	-	99.0, CH	5.94, d (8.2)
6	131.3, CH	7.13, d (0.5)	131.6, CH	7.15, s	132.8, CH	7.52, d (8.2)
7	45.4, CH	2.29, t (10.1)	45.3, CH	2.26, t (10.1)	45.2, CH	2.46, t (10.8)
8	50.9, CH	1.54, ddd (23.9, 10.2, 2.6)	50.9, CH	1.53, ddd (24.0, 10.2, 2.6)	48.1, CH	3.00, m
9α	38.5, CH_2_	0.89, dd (23.9, 12.1)	38.5, CH_2_	0.89, dd (24.0, 12.0)	43.6, CH_2_	1.73, m
9β		1.89, *br* d (14.9)	-	1.87. *br* d (12.2)	-	0.89, m
10	34.1 CH	1.70, m	34.1, CH	1.68, m	33.0, CH	1.61, m
11α	47.1, CH_2_	1.07, dd (24.8, 11.8)	47.1, CH_2_	1.06, dd (24.7, 11.8)	45.9, CH_2_	1.71, m
11β	-	1.80, *br* d (13.2)	-	1.78, *br* d (13.0)	-	0.77, m
12	41.9, CH	1.75, m	41.9, CH	1.71, m	33.0, CH	2.37, m
13	79.7, CH	3.72, m	79.7, CH	3.68, m	144.7, CH	5.53, ddd (16.8, 10.0, 9.2)
14	19.1, CH_3_	1.31, d (6.3)	19.1, CH_3_	1.28, d (6.2)	113.0, CH	4.75, dd (16.5, 2.1), 4.61 dd (10.0, 2.1)
15	22.8, CH_3_	1.02, d (6.6)	22.8, CH_3_	0.99, d (6.4)	23.2, CH_3_	0.92, d (6.6)
16	23.2, CH_3_	1.15, d (6.8)	23.2, CH_3_	1.12, d (6.7)	21.0, CH_3_	0.71, dd (6.7)
17	126.6, C		129.3, C	-	-	-
18/22	131.3, CH	7.25, d (8.8)	131.2, CH	7.33, d (8.7)	-	-
19/21	115.9, CH	6.80, d (8.8)	117.9, CH	7.13, d (8.7)	-	-
20	157.9, C	-	158.1, C	-	-	-
Ribose	-	-	-	-	-	-
1	-	-	102.4, CH	5.64, d (4.4)	-	-
2	-	-	73.4, CH	4.19, dd (6.4, 4.5)	-	-
3	-	-	71.2, CH	4.10, dd (6.5, 3.2)	-	-
4	-	-	87.5, CH	5.15, dd (7.0, 3.5)	-	-
5a	-	-	63.2, CH_2_	3.65, dd (12.1, 4.0)	-	-
5b	-	-	-	3.71, dd (12.1, 3.4)	-	-

^a^ Recorded at 125 MHz; ^b^ carbon type inferred from DEPT (Distortionless Enhancement by Polarization Transfer) and HMQC (Heteronuclear Multiple Quantum Correlation) experiments; ^c^ recorded at 500 MHz.

Compound **2** was isolated as a brown powder. The molecular formula was determined to be C_26_H_33_NO_7_ by analysis of the HR-TOF-MS ion peak at *m/z* 494.2131 [M + Na]^+^ (calcd. 494.2149). The IR spectrum suggested the presence of hydroxyl (3251 cm^−1^) and aromatic (1612 cm^−1^) groups. ^1^H and ^13^C NMR spectra showed signals in close agreement with those of Compound **1**, except that the OH group was replaced by a sugar moiety. The diastereotopic nature of the protons in the ^1^H NMR of Compound **2** suggested that the introduction of the sugar moiety significantly hinders rotation around the aryl-pyridone bond. Analysis of the 1D and 2D NMR data and comparison with those of Compound **1** led to identification of the structure of **2** as a glycosylated derivative of **1** with ribofuranose [22]. The glycosylated position in the aglycone was deduced to be at C-20 by HMBC experiments (Supplementary Information, Figures S18 and S19). The relative configuration of the aglycon of **2** was proved to be the same as compound **1** after detailed analysis of the proton coupling constants of **2**. The configuration of the glycosidic linkage of the ribofuranoside moiety in **2** was determined to be β on the basis of the *J* value (*δ*_H_ 5.64 (d, *J* = 4.4 Hz)) of the anomeric proton. Therefore, the structure of **2** was elucidated as a new ribofuranoside of **1**, and given the trivial name, trichodin B. The assignment of the NMR signals of **2** is listed in Table 1.

### 2.3. Biological Activities

Compound **1** showed moderate antibiotic activities against the Gram-positive *Bacillus subtilis* with an IC_50_ value of 27.05 ± 0.53 µM, *Staphylococcus epidermidis* with anIC_50_ value of 24.28 ± 3.90 µM, *Staphylococcus aureus* (MRSA) with an IC_50_ value >80 μM and the yeast, *Candida albicans* (IC_50_ value 25.38 ± 0.41 μM). No activity was observed against *Trichophyton rubrum*. While Compound **2** exhibited no antimicrobial effects against all test strains, Compound **3** was active against *B. subtilis*, *S. epidermidis*, *Staphylococcus aureus* (MRSA), *C. albicans* and *Trichophyton rubrum* with IC_50_ values of 5.28 ± 0.42 μM, 4.25 ± 0.81 μM, 4.40 ± 0.14 μM, 26.25 ± 0.14 μM and 4.05 ± 1.28 μM, respectively.

## 3. Experimental Section

### 3.1. General Experimental Procedures

Optical rotations were recorded on a Perkin Elmer 241 polarimeter. The IR spectra were run on a Perkin Elmer spectrometer with an ATR unit. ^1^H NMR (500 MHz) and ^13^C NMR (125 MHz) spectra were measured at 25 °C on a Bruker AVANCE DRX 500 NMR spectrometer with TMS as the internal standard. The signals of the residual solvent protons and the solvent carbons were used as internal references (δ_H_ 3.31 ppm and δ_C_ 49.0 ppm for methanol-*d*_4_). High-resolution mass spectra were acquired on a benchtop time-of-flight spectrometer (micrOTOF II, Bruker Daltonics, Bremen, Germany) with positive electrospray ionization (ESI).

Analytical reversed phase HPLC-DAD(UV)-MS experiments were performed using a C_18_ column (Phenomenex Onyx Monolithic C18, 100 × 3.00 mm) and applying an H_2_O/acetonitrile (ACN) gradient with 0.1% formic acid added to both solvents (gradient: 0 min 5% ACN, 4 min 60% ACN, 6 min 100% ACN; flow: 2 mL/min) on a VWR Hitachi Elite LaChrom system with an L-2450 diode array detector, an L-2130 pump and an L-2200 autosampler. This HPLC system was coupled to an ESI-ion trap detector with positive ionization (Esquire 4000, Bruker Daltonics) for mass detection.

The preparative HPLC was conducted with a VWR HPLC-UV system (VWR International LaPrep, VWR, Darmstadt, Germany) equipped with a pump (P110), a UV detector (P311), a Smartline 3900 autosampler (Knauer, Berlin, Germany), a LABOCOL Vario-2000 fraction collector (LABOMATIC, Weil am Rhein, Germany) and a Phenomenx Gemini-NX column (C18, 10 μm, 110 Å, 100 × 50 mm). An H_2_O/acetonitrile (ACN) gradient with 0.1% formic acid added to both solvents was applied (gradient: 0 min 10% ACN with a flow of 40 mL/min; 0.5 min 10% ACN, 17 min 60% ACN, 22 min 100% ACN, 26 min 10% ACN; flow: 100 mL/min).

Semi-preparative HPLC was carried out using an HPLC-UV system (VWR Hitachi Elite LaChrom system, VWR, Darmstadt, Germany) consisting of an L-1230 pump, an L-2450 diode array detector, an L-2200 autosampler and a Phenomenex Luna column (Silica (2), 5 µm, 100 Å, 250 × 10 mm), respectively.

### 3.2. Isolation, Cultivation, Identification and Storage of the Producer Strain, MF106

Strain MF106 was isolated from a Greenland Sea (Fram Strait) sample taken on the cruise FS “Polarstern” ARKTIS VIII/1 in June, 1991 [23], and cultured on WSP30 medium, a modified Wickerham-medium, which consisted of 1% glucose, 0.5% peptone, 0.3% yeast extract, 0.3% malt extract and 3% sodium chloride (pH = 6.8) [24]. The strain was stored at the Kultursammlung Mariner Pilze (KSMP-Kiel, Kiel, Germany) using two methods, liquid nitrogen and the Microbank System at −80 °C (MAST DIAGNOSTIKA, Reinfeld, Germany).

The identification has been performed by morphological criteria using scanning electron microscopy. Colonies growing on WSP30 agar plates were cut in 1 cm² samples, transferred through an ethanol series (30%, 50%, 70%, 90%, 3 × 100%; each 15 min) and subsequently critical-point-dried in liquid carbon dioxide (Balzers CPD030, Oerlikon Balzers Coating Germany GmbH, Bingen am Rhein, Germany). Samples were sputter-coated with gold-palladium (Balzers SCD004, Oerlikon Balzers Coating Germany GmbH) and analyzed with a ZEISS DSM 940 scanning electron microscope (ZEISS, Oberkochen, Germany).

### 3.3. Fermentation and Production of Extracts

Strain MF106 was cultured on WSP30 agar plates at 26 °C for 15 days. This pre-culture was used for the inoculation of 12 × 2-L Erlenmeyer flasks containing 750 mL WSP30TM medium (1% glucose, 0.5% peptone, 0.3% yeast extract, 0.3% malt extract and 3% tropic marine salt (pH = 6.8)) each. After incubation for 20 days at 28 °C in the dark as static cultures, extracts of the cultures were obtained. The mycelium was separated from the culture broth. One hundred fifty milliliters of ethanol were added to the mycelium of each flask and homogenized. After a centrifugation step at 10,000 rpm for 10 min, the supernatant was collected, and the ethanol was removed by evaporation. The remaining aquatic phases of all 12 flasks were combined and extracted twice with 100 mL ethyl acetate. The organic phase was used for evaporation. The resulting residue was dissolved in 5 mL methanol to get the extract of the mycelium. The fermentation broth was extracted with ethyl acetate (200 mL per each flask). One hundred milliliters of deionized water were added to the organic phase. The upper phases of all flasks were combined and used for evaporation. The residue was dissolved in 5 mL methanol to get the extract of the culture broth. Both extracts were subjected to analytical HPLC-UV/MS. For the purification of Compounds **1**–**3**, both extracts were combined.

### 3.4. Isolation of Compounds

The extract was fractionated with the VWR LaPrep HPLC-UV system. Eleven fractions were obtained; whereas Fraction 8 contained Compound **1**; Fraction 4 contained Compound **2**; and Fractions 6 and 7 contained Compound **3**. Fraction 4 and 8 were further purified by the VWR Hitachi Elite LaChrom system applying the following parameters for Compound **1** (gradient: 0 min 43% ACN, 20 min 44% ACN; flow 15 mL/min; UV detection at 261 nm; *t*_R_ 8.2 min) and for Compound **2 **(gradient: 0 min 34% ACN, 20 min 42% ACN; flow 15 mL/min; UV detection at 210 nm; *t*_R_ 6.2 min) The yields for Compounds **1**–**3** were 2.3 mg, 1.2 mg and 52.9 mg, respectively.

Trichodin A (**1**): brown powder; [α]^24^_D_ −54 (*c* 0.1, MeOH); UV (MeOH) λ_max_ (log *ε*) 202 (4.45), 232 (4.26) nm; IR ν_max_: 3105, 2952, 1614, 1547, 1513, 1417, 1263, 1239, 1212, 1043, 894, 830, 524 cm^−1^; ^1^H NMR and ^13^C NMR, see Table 1; ESIMS *m/z* 362 [M + Na]^+^; HR-TOF-MS *m/*z 362.1751 [M + Na]^+^ (calcd. for C_21_H_25_NNaO_3_, 362.1727).

Trichodin B (**2**): brown powder; [α]^24^_D_ −46 (*c* 0.1, MeOH); UV (MeOH) λ_max_ (log *ε*) 203 (4.30), 234 (4.21) nm; IR ν_max_: 3251, 2926, 1639, 1612, 1510, 1435, 1378, 1230, 1042, 833 cm^−1^; ^1^H NMR and ^13^C NMR, see Table 1; ESI, MS *m/z* 494 [M + Na]^+^; HR-TOF-MS *m/*z 494.2131 [M + Na]^+^ (calcd. for C_26_H_33_NNaO_7_, 494.2149).

### 3.5. Antimicrobial Activity Assays

The antimicrobial activities of Compounds **1**–**3** against *Bacillus subtilis* (DSM 347) and the human pathogenic yeast, *Candida albicans* (DSM 1386), were determined according to Ohlendorf *et al*. [25].The bioassays using the clinically relevant bacterial strains, *Staphylococcus epidermidis* (DSM 20044) and methicillin-resistant *Staphylococcus aureus* (MRSA) (DSM 18827) were performed as described by Silber *et al*. [26]. *Trichophyton rubrum*, a dermatophyte, was tested according to Jansen *et al.* [27].

## 4. Conclusions

In addition to the known activities of pyridoxatin, *i.e.*, the inhibition of lipid peroxidation induced by free radicals, the inhibition of the hemolysis of rat erythrocytes, the inhibition of the growth of HeLa cells and *Candida albicans*, which were described by Teshima *et al*. [9], antimicrobial effects against further human pathogenic strains, such as methicillin-resistant *Staphylococcus aureus* (MRSA), were observed in this study.

This is the first example of a pyridone with a mono sesquiterpene being glycosided. Trichodin A and trichodin B are structurally interesting, which would provide opportunities to design and synthesize new analogs that could improve the antibiotic activity of these new compounds.

## Supplementary Files

Supplementary File 1 Supplementary Information (PDF, 928 KB)
