# Supplementary material for: Two New Antibiotic Pyridones Produced by a Marine Fungus, Trichoderma sp. Strain MF106
Source: Mar Drugs. 2014 Mar 6;12(3):1208–19. doi: 10.3390/md12031208 (PMC3967205; doi:10.3390/md12031208)
Supplement: Supplementary File 1 — Supplementary Information (PDF, 928 KB) [file marinedrugs-12-01208-s001.pdf]

Supplementary Information

Figure S1. ^{1}H NMR in CD_3OD for compound **1**.
Figure S2. ^{1}H NMR-2 in CD_3OD for compound **1**.
Figure S3. ^{13}C NMR in CD_3OD for compound **1**.
Figure S4. COSY in CD_3OD for compound **1**.
Figure S5. COSY-2 in CD_3OD for compound **1**.
Figure S8. HSQC in CD_3OD for compound **1**.
Figure S7. HSQC-2 in CD_3OD for compound **1**.
Figure S8. HMBC in CD_3OD for compound **1**.
Figure S9. HMBC-2 in CD_3OD for compound **1**.
Figure S10. NOESY in CD_3OD for compound **1**.
Figure S11. NOESY-2 in CD_3OD for compound **1**.
Figure S12. ^{1}H NMR in CD_3OD for compound **2**.
Figure S13. ^{1}H NMR-2 in CD_3OD for compound **2**.
Figure S14. ^{13}C NMR in CD_3OD for compound **2**.
Figure S15. COSY in CD_3OD for compound **2**.
Figure S16. HSQC in CD_3OD for compound **2**.
Figure S17. HSQC-2 in CD_3OD for compound **2**.
Figure S18. HMBC in CD_3OD for compound **2**.
Figure S19. HMBC-2 in CD_3OD for compound **2**.
Figure S20. HR-TOF-MS in MeOH for compound **1**.
Figure S21. HR-TOF-MS in MeOH for compound **2**.

Figure S1. ^{1}H NMR in CD_3OD for compound **1**.

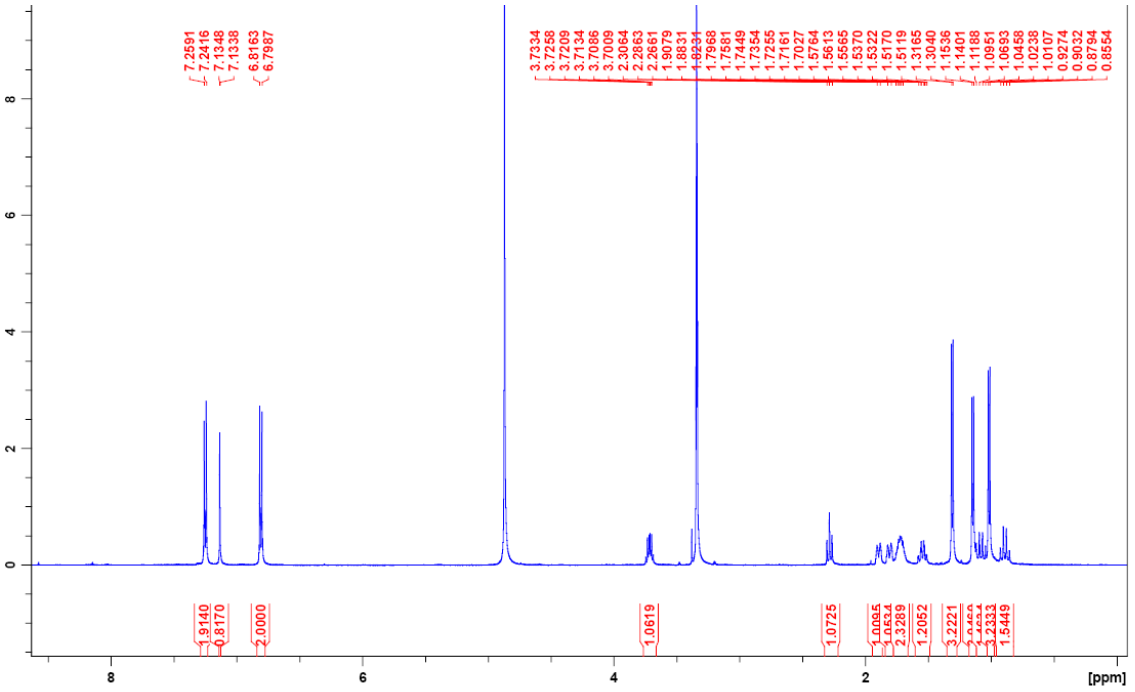

Figure S2. ^{1}H NMR-2 in CD_3OD for compound **1**.

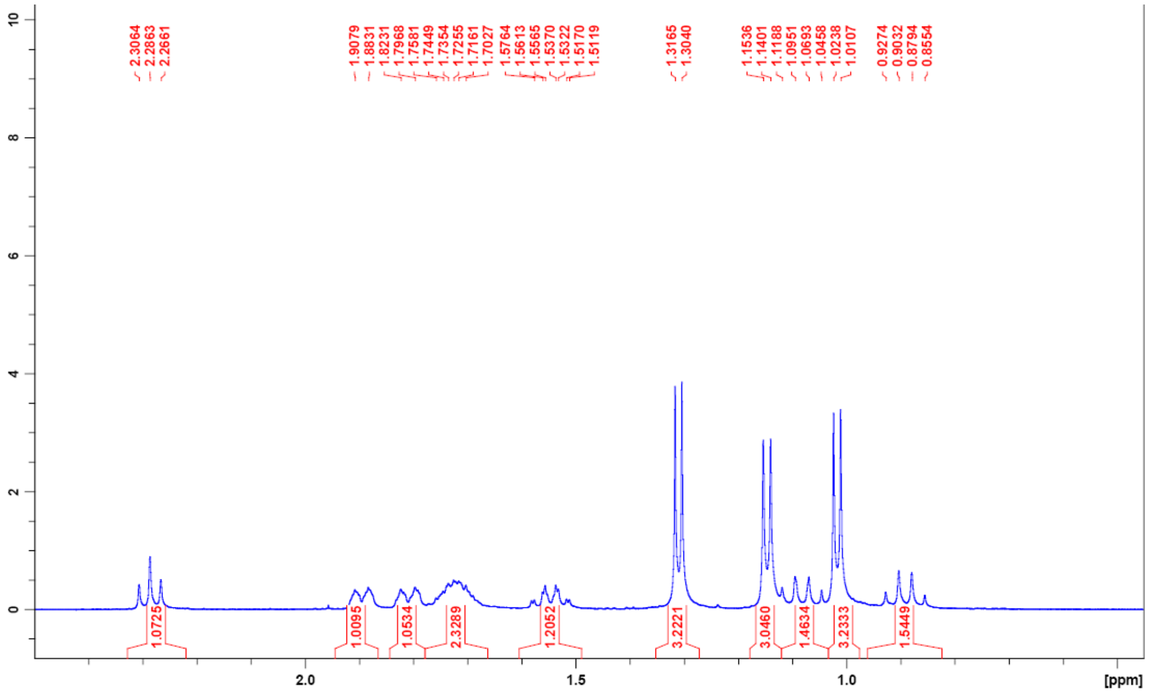

Figure S3. ^{13}C NMR in CD_3OD for compound **1**.

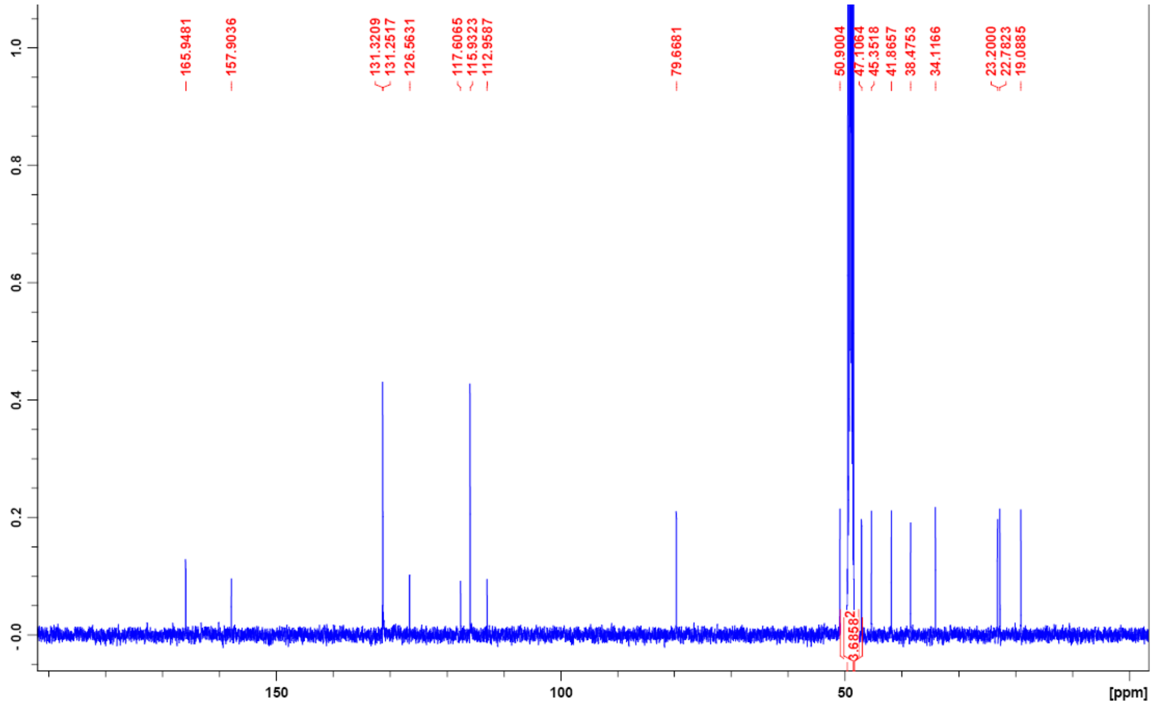

Figure S4. COSY in CD_3OD for compound **1**.

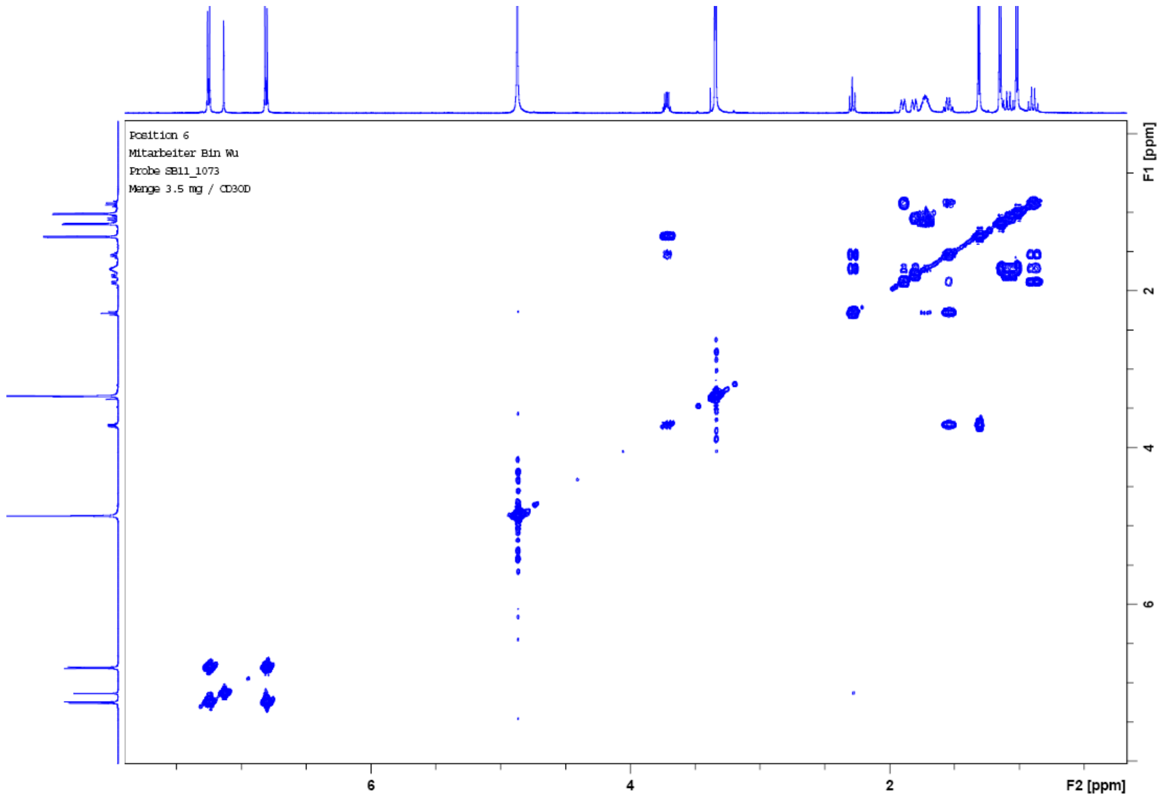

Figure S5. COSY-2 in CD_3OD for compound **1**.

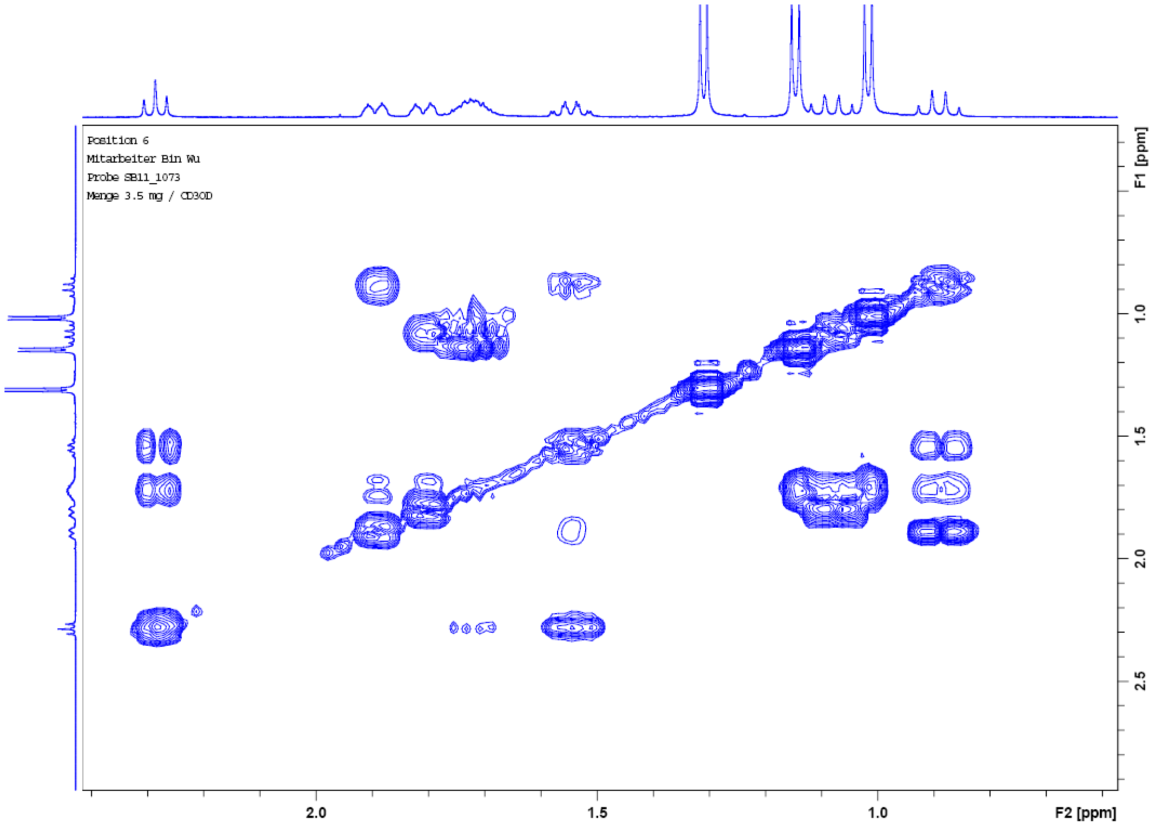

Figure S6. HSQC in CD_3OD for compound **1**.

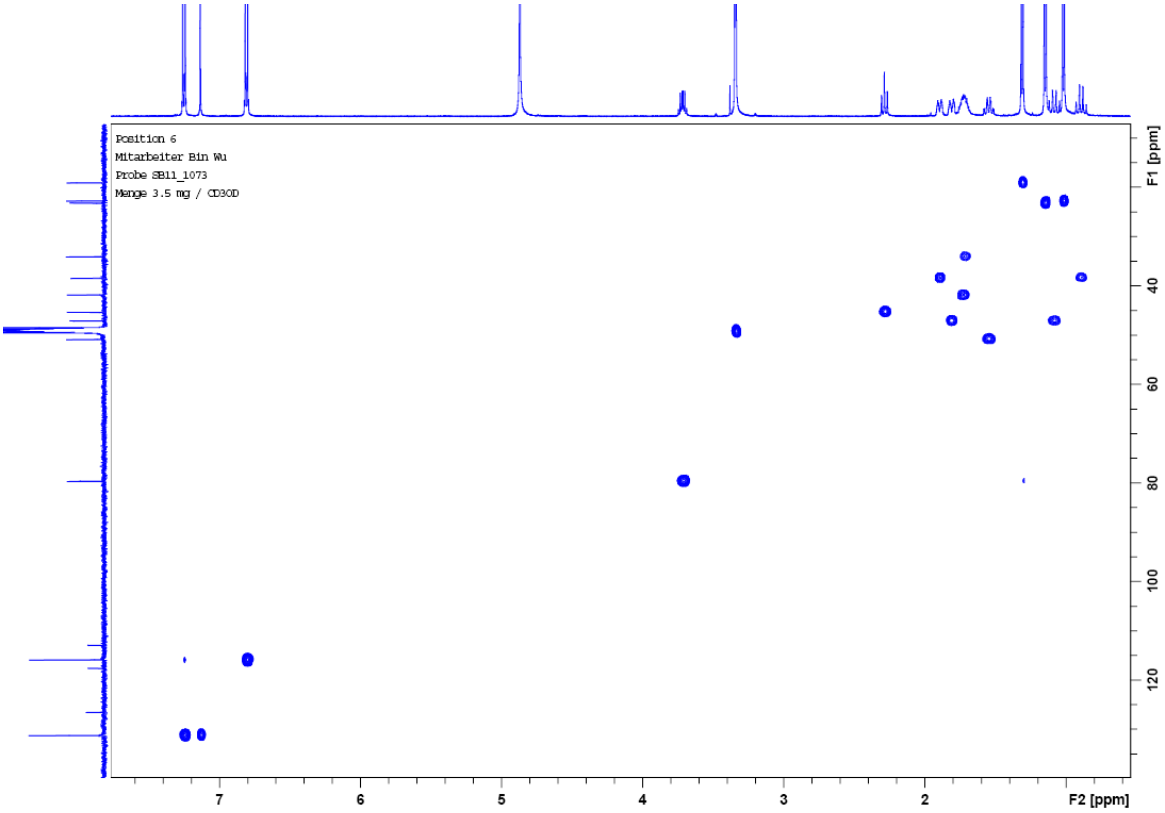

Figure S7. HSQC-2 in CD_3OD for compound **1**.

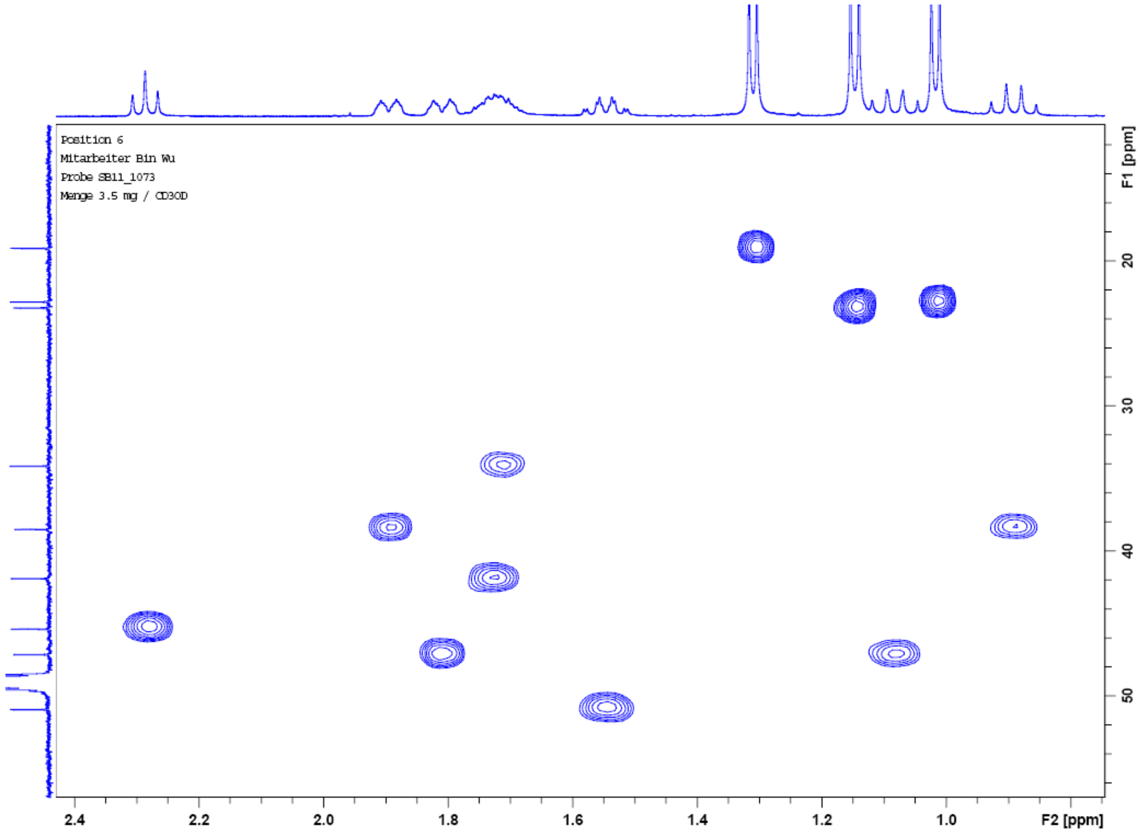

Figure S8. HMBC in CD_3OD for compound **1**.

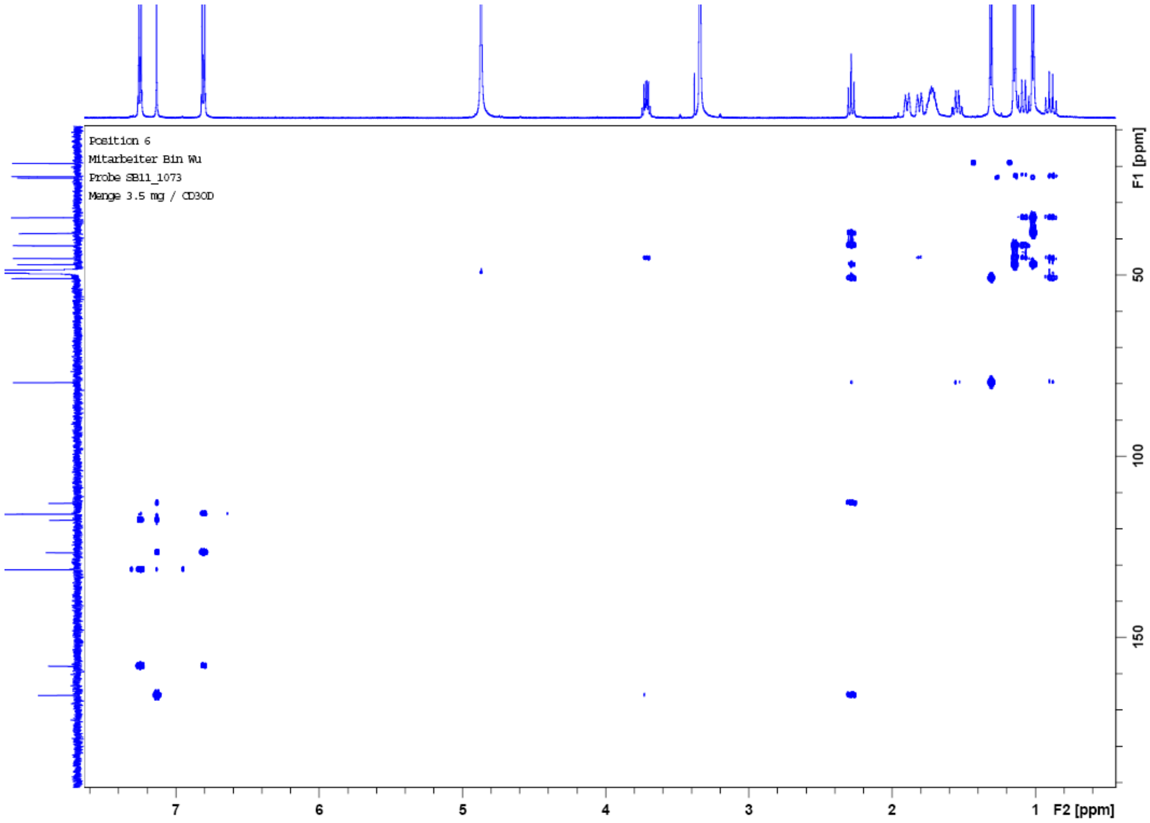

Figure S9. HMBC-2 in CD_3OD for compound **1**.

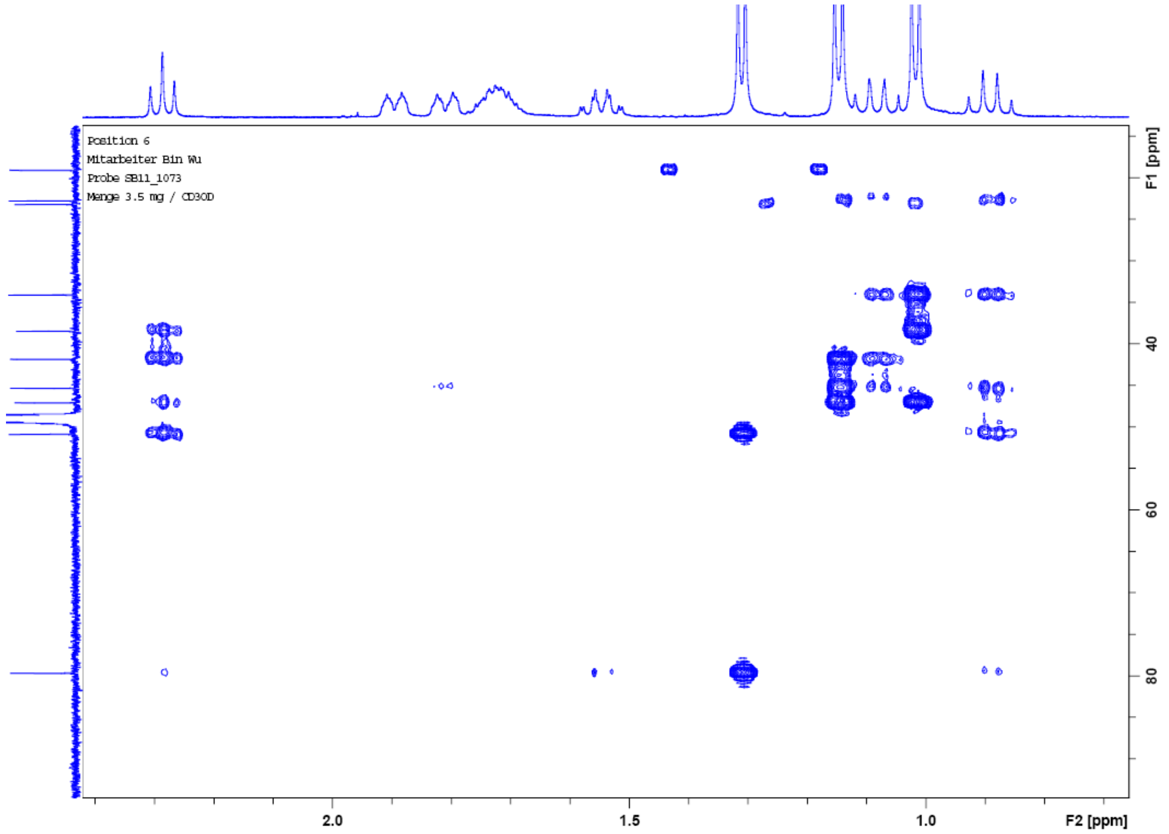

Figure S10. NOESY in CD_3OD for compound **1**.

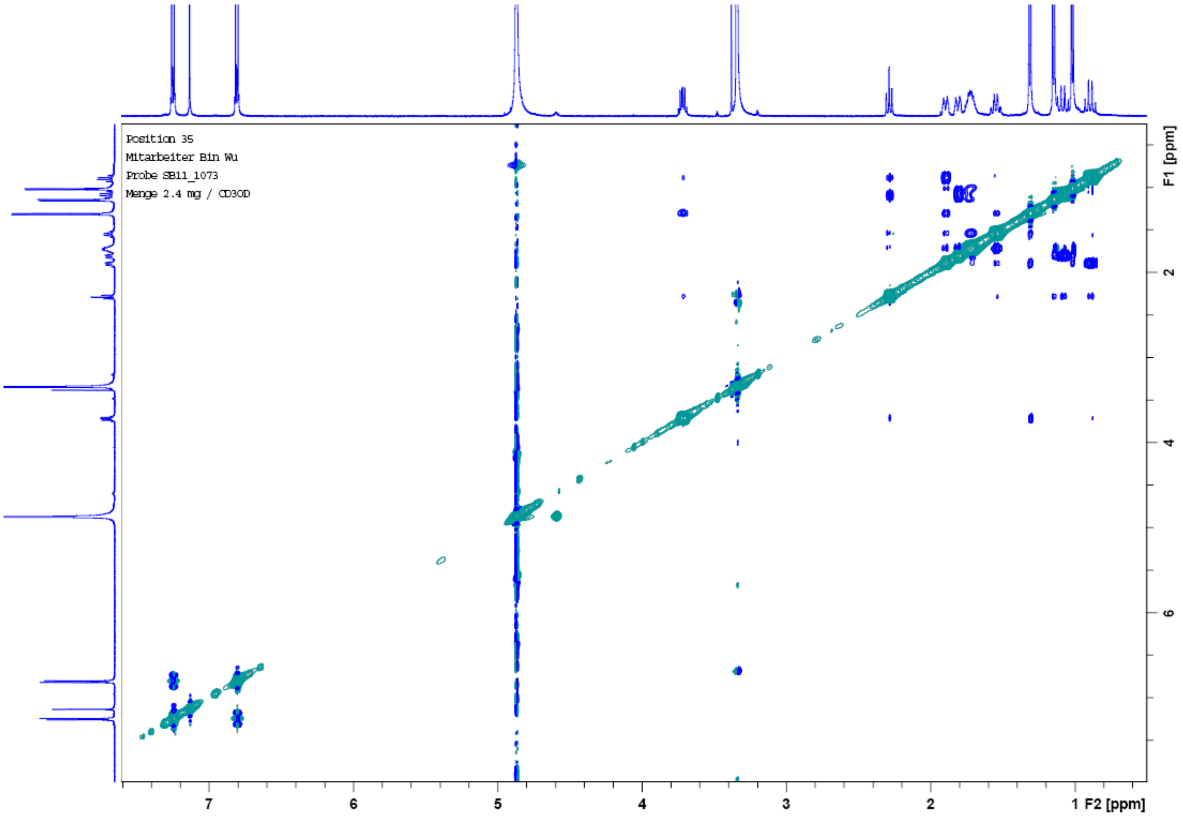

Figure S11. NOESY-2 in CD_3OD for compound **1**.

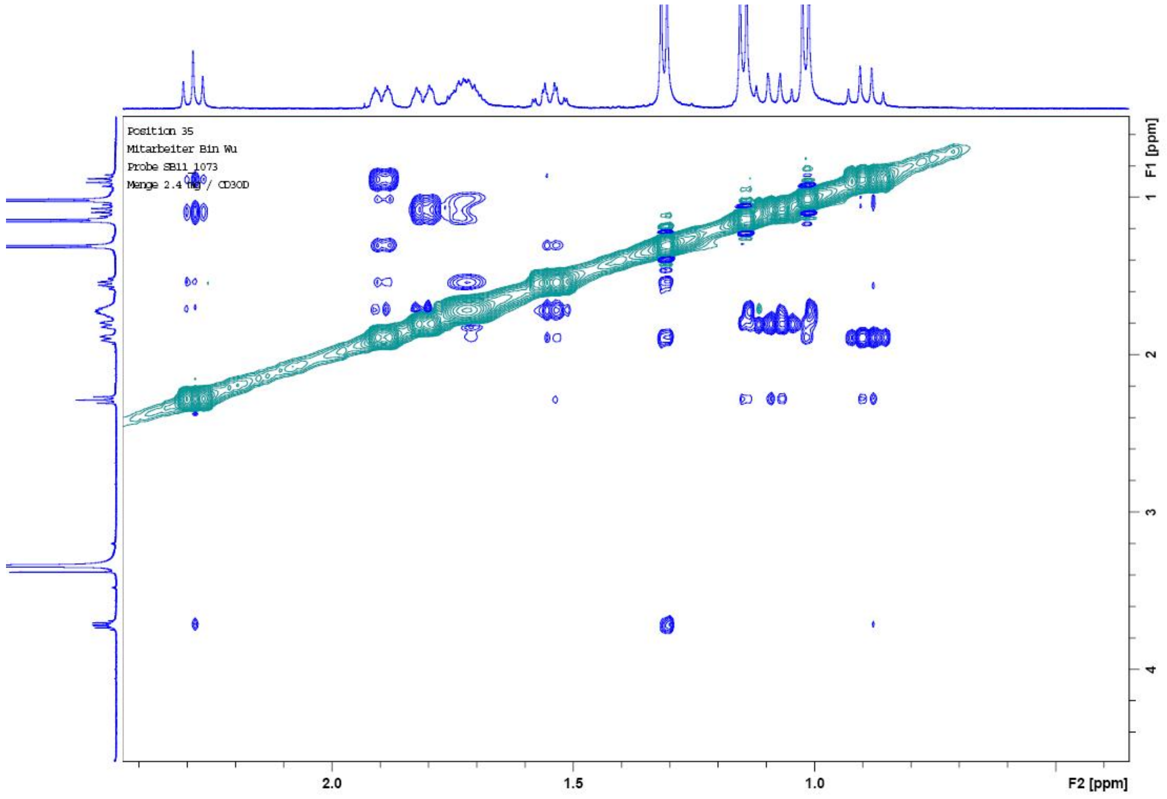

Figure S12. ^{1}H NMR in CD_3OD for compound **2**.

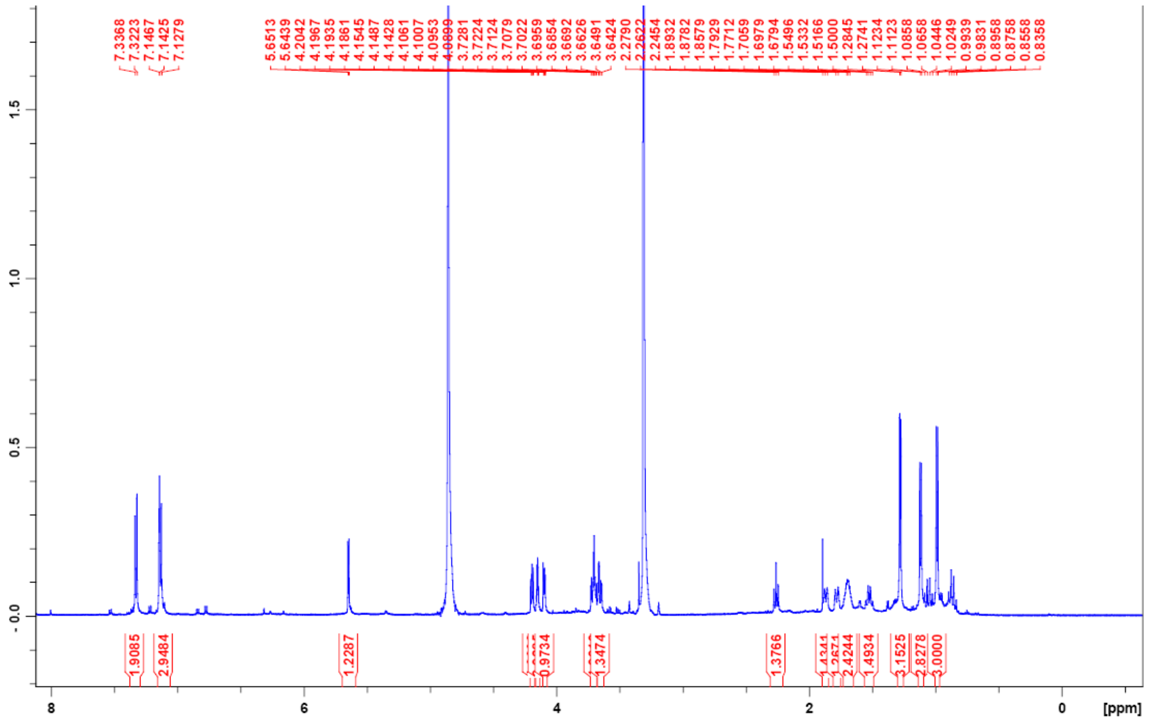

Figure S13. ^{1}H NMR-2 in CD_3OD for compound **2**.

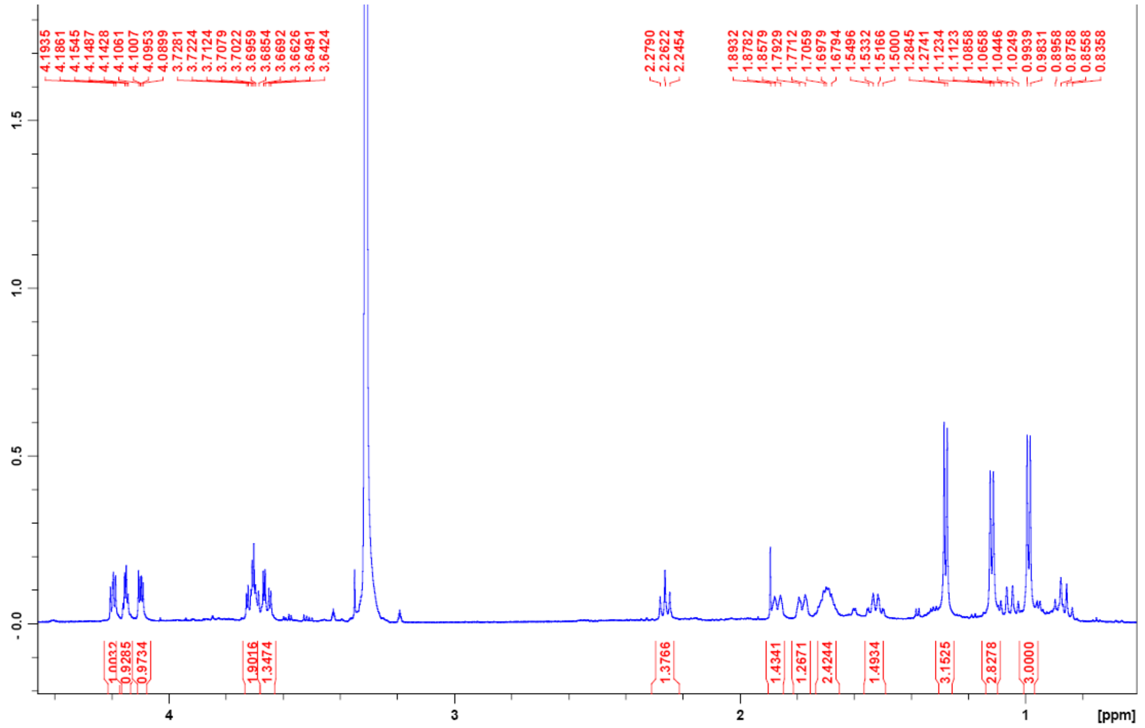

Figure S14. ^{13}C NMR in CD_3OD for compound **2**.

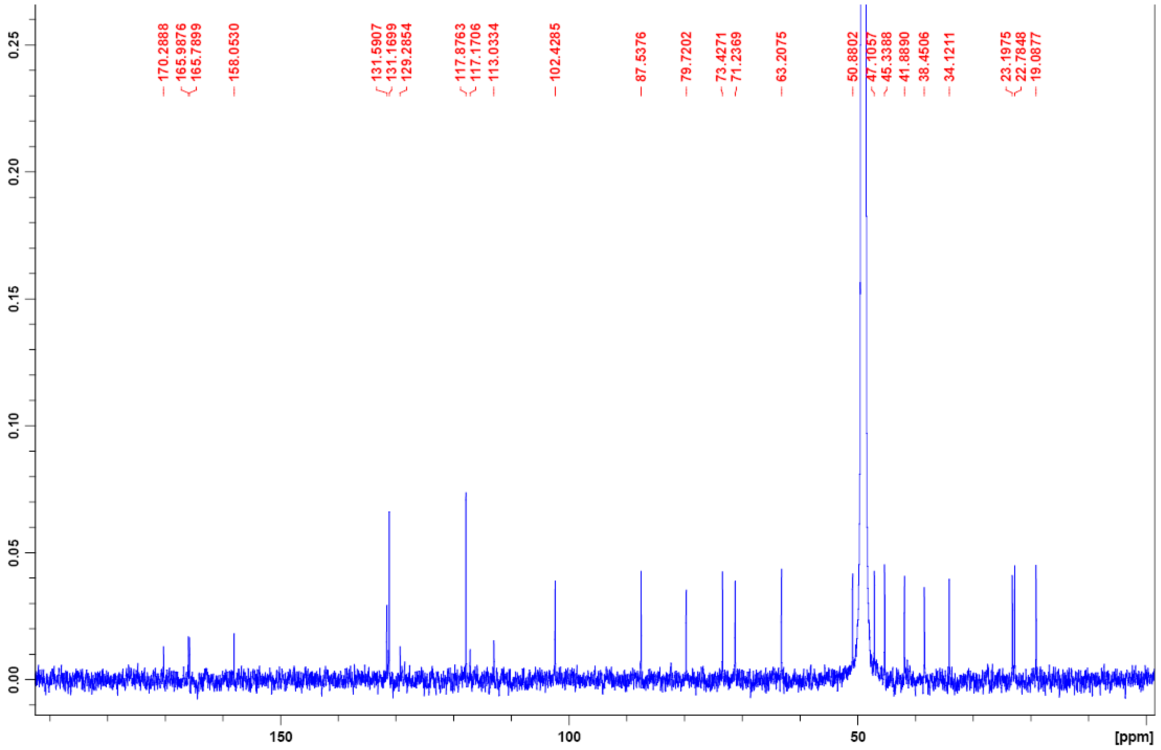

Figure S15. COSY in CD_3OD for compound **2**.

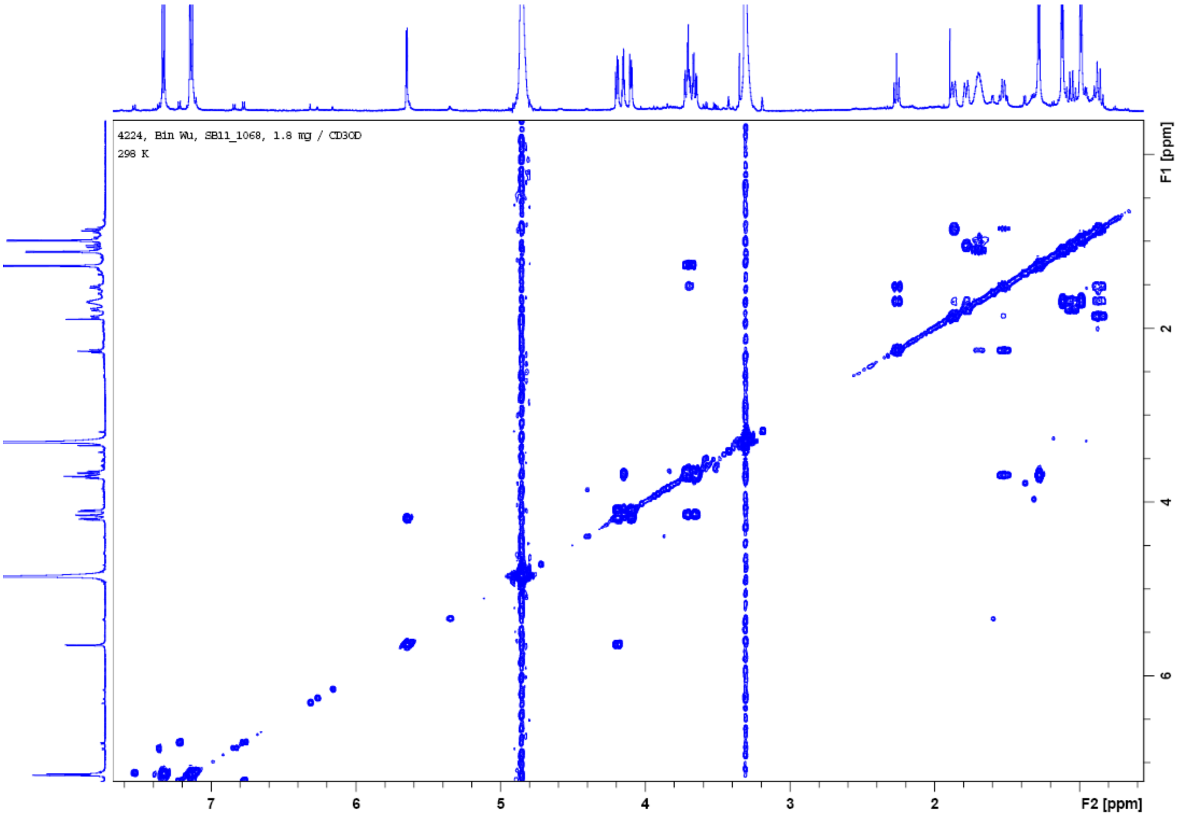

Figure S16. HSQC in CD_3OD for compound **2**.

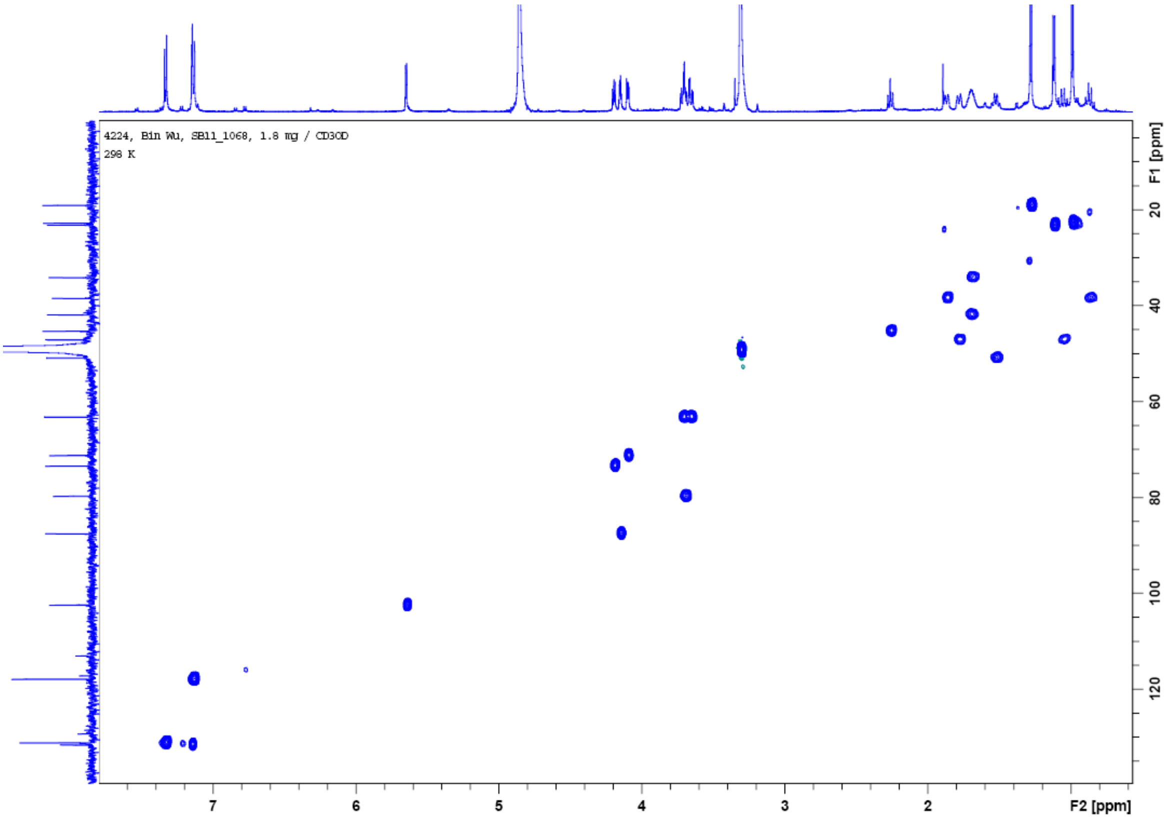

Figure S17. HSQC-2 in CD_3OD for compound **2**.

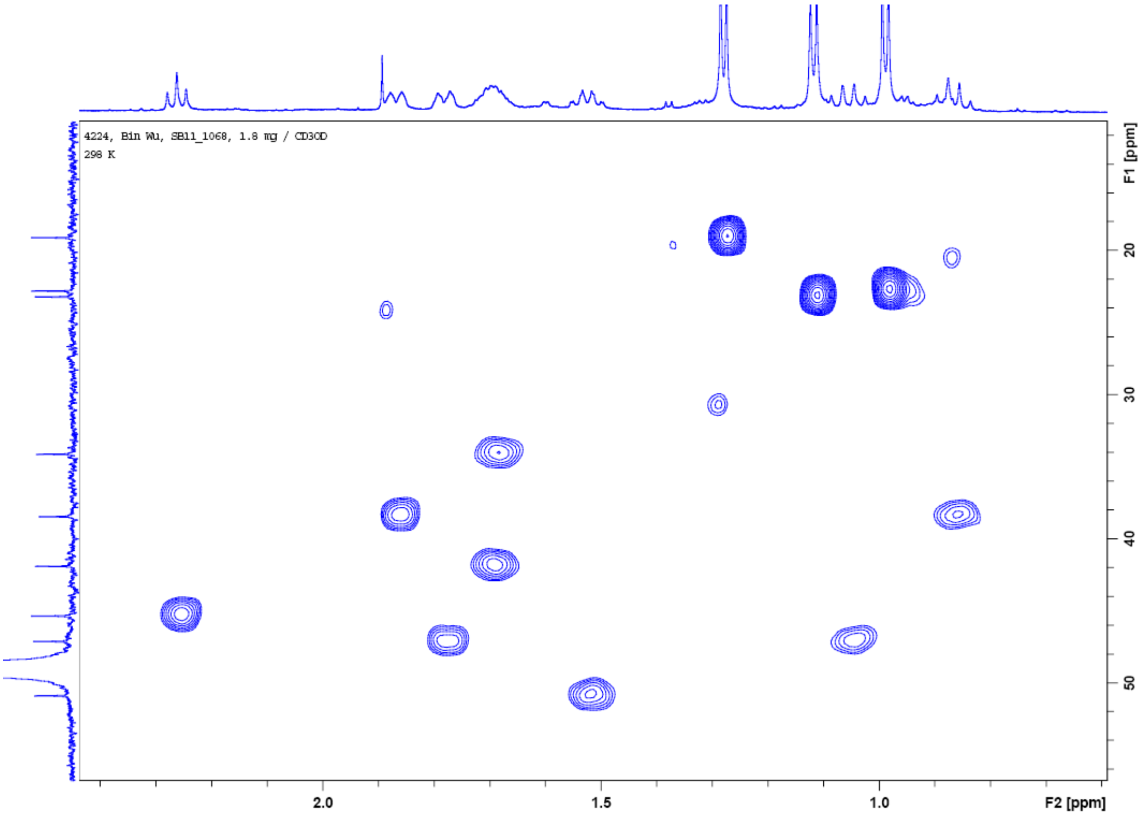

Figure S18. HMBC in CD_3OD for compound **2**.

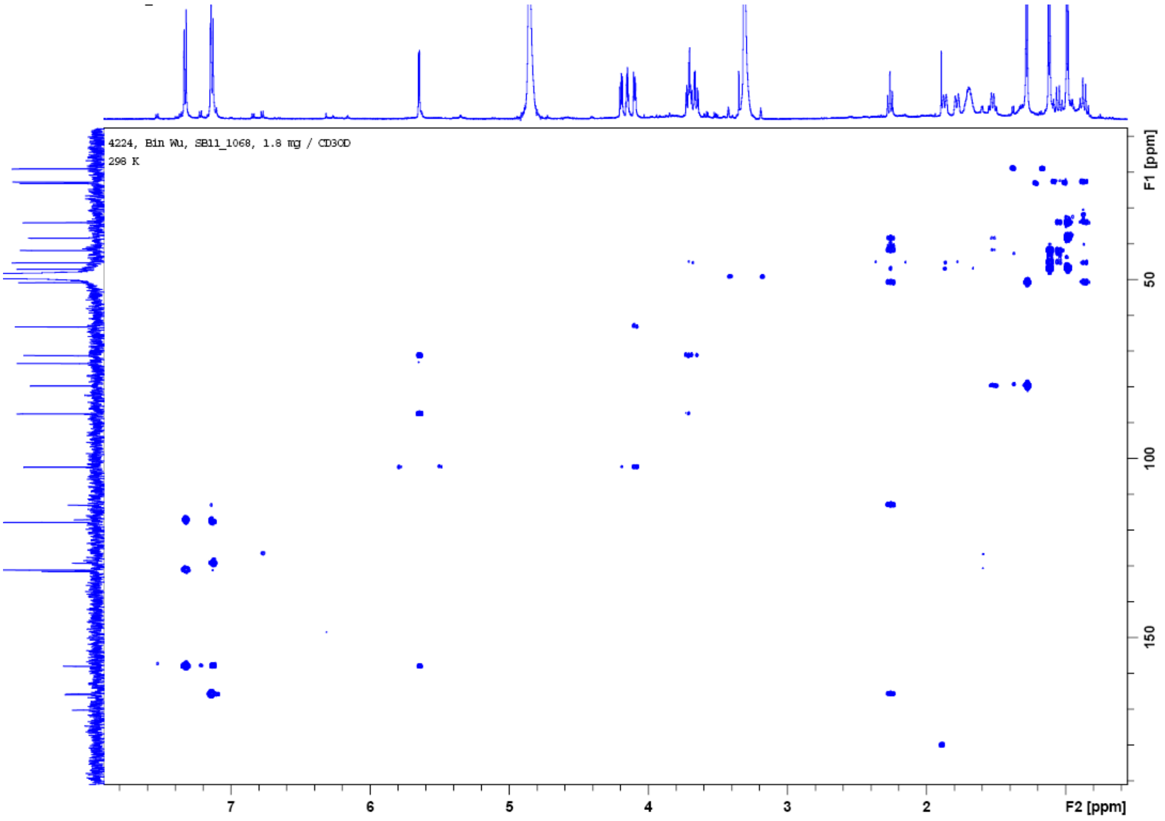

Figure S19. HMBC-2 in CD_3OD for compound **2**.

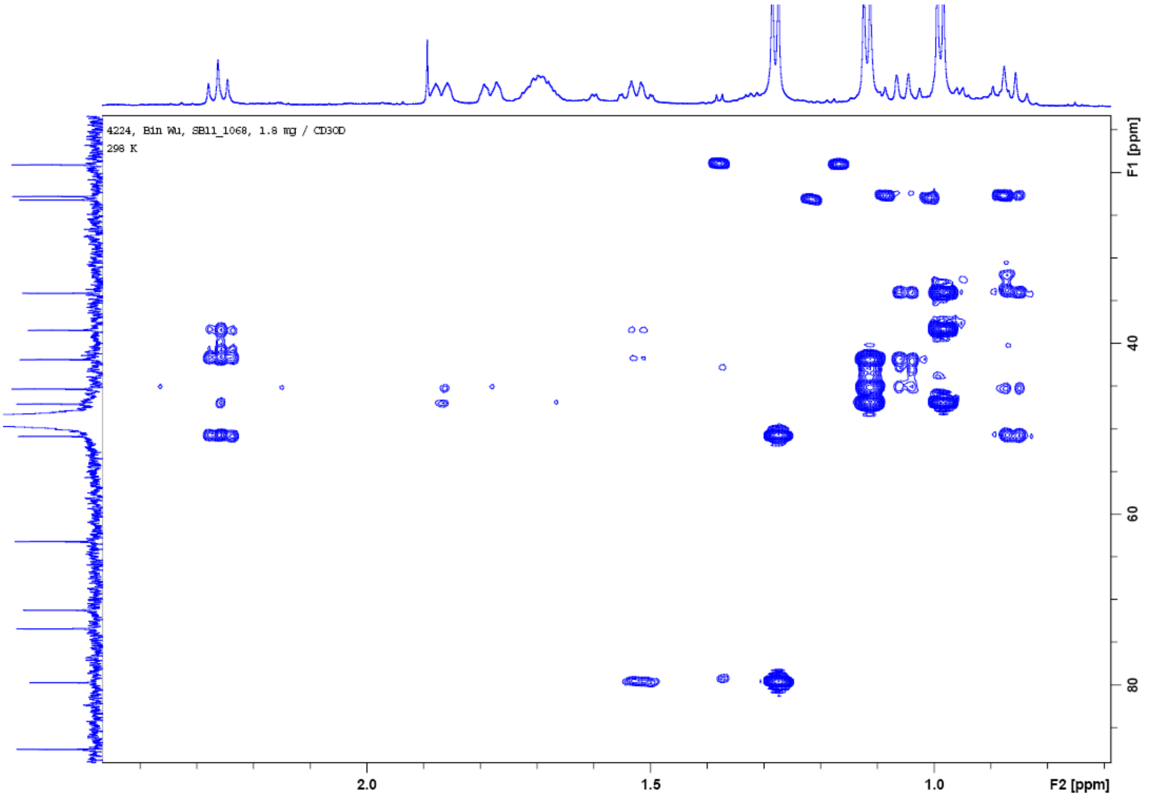

Figure S20. HR-TOF-MS in MeOH for compound **1**.

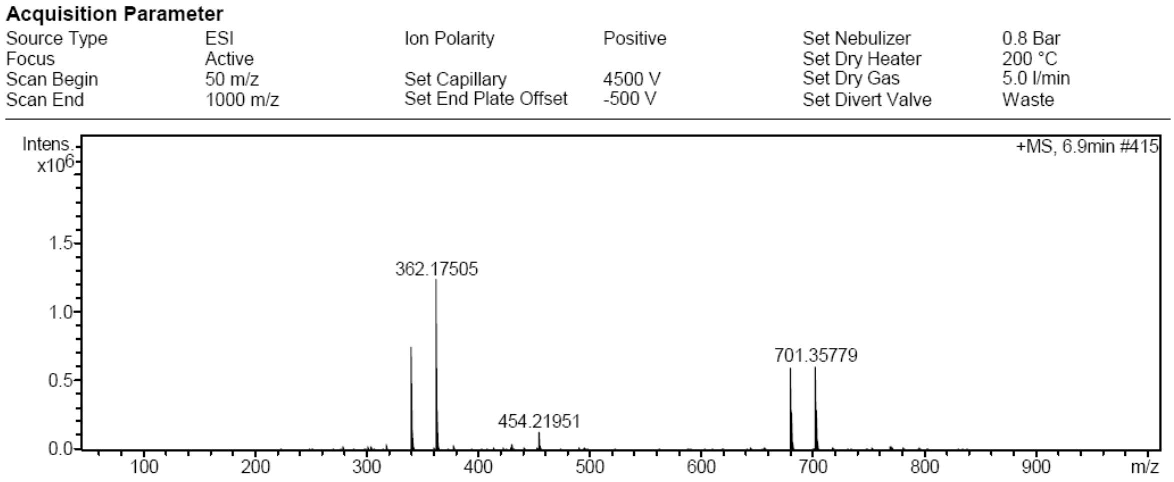

Figure S21. HR-TOF-MS in MeOH for compound **2**.

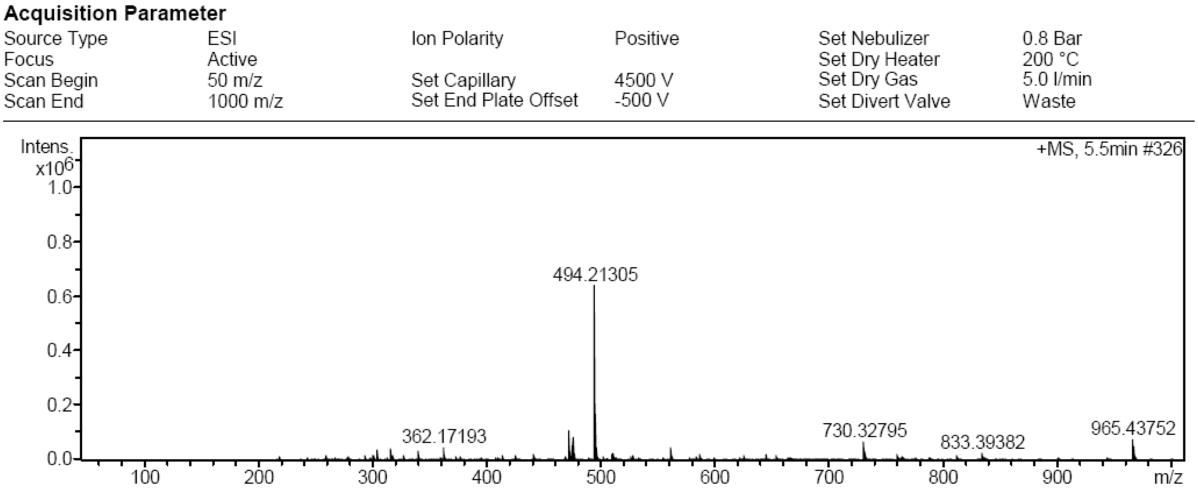

© 2014 by the authors; licensee MDPI, Basel, Switzerland. This article is an open access article distributed under the terms and conditions of the Creative Commons Attribution license (http://creativecommons.org/licenses/by/3.0/).
